# COVID-19 epidemic response in Ghana

**DOI:** 10.4314/gmj.v54i4s.2

**Published:** 2020-12

**Authors:** George Amofah

**Affiliations:** President, Ghana Public Health Association george.amofah@ghsmail.org

The year 2020 has looked like a fairy tale as the COVID-19 pandemic swept across the world with devastating socio-economic and health consequences. The impact of the pandemic has depended, largely, on preparedness and response of countries, and their ability to adjust to the fast-evolving pandemic. The World Health Organization (WHO) declared the novel coronavirus outbreak a public health emergency of international concern (PHEIC) on 30th January 2020, and Ghana reported its first two confirmed cases on 12^th^ March 2020. The President imposed a partial lockdown on Greater Accra, Greater Kumasi, and parts of Kasoa district on 28^th^ March 2020 for an initial two weeks, which was extended further to three weeks before unlocking. I have been privileged to be part of the national response since March 2020 to date, and I share my impressions on what has worked, and challenges faced so far. A major success factor has been the availability of a national blueprint to develop a COVID-19 Pandemic Preparedness Plan based on earlier experiences with Influenza H1N1, SARS and Ebola outbreaks. The blueprint set out the governance system for responding to pandemics, including establishing an Emergency Operations Centre and a National Technical Coordinating Committee. A high-level COVID-19 coordinator, in the person of an experienced retired Deputy Director-General of WHO and a supra coordinating committee chaired by the President of the Republic himself, were also established. This governance system has been key to Ghana's success in responding to the pandemic as it ensured that the emergency was tackled through a onehealth, one-nation approach. Initially, there was confusion and lack of clarity about the roles and responsibilities of the various structures leading to delays in dealing with emerging situations.

Due to the rapid spread of the virus globally, capacity and logistics to deal with the situation lagged. The policy of Track, Test and Treat that was instituted was very helpful as systems were developed first to identify, track, and test all suspected cases. The test employed was the reverse transcriptase real-time polymerase chain reaction for the viral antigen. Depending on test results, they were either quarantined, isolated, or admitted for management in designated facilities.

A major challenge was the inadequate capacity to test samples which led to a backlog of untested samples for weeks with resultant increased anxiety and criticism by healthcare workers and those tested.

Indeed, the initial enhanced tracking and testing approach that was adopted enabled many secondary contacts to be identified, increasing the testing rate; this approach also contributed to the backlog of samples and in the delay in reporting results. The gradual improvement in PCR-testing facilities from an initial 2 to over 15 spread across the country helped to relieve the situation. The “pooling” methodology adopted by the Noguchi Memorial Institute for Medical Research, the leading testing facility, rapidly dealt with the testing congestion and proved to be costeffective. Amidst challenges with the supply of reagents and few testing sites, the approach had to be amended to focus mostly on contacts of positive cases later. The drone technology and acquisition of over 300 ambulances before the pandemic were positive additions to strengthening the health system, which became handy during the pandemic, as samples could be transported quickly to testing sites from afar. There has been a major challenge with identifying and equipping quarantine, isolation, and treatment centres and it is a credit to the health system, with support from the local government system, that this major challenge was largely addressed.

There has been very poor adherence to recommended safety protocols partly due to non-compliance by politicians and even by health workers. Risk communication has been problematic throughout the pandemic, and this attitude must be addressed moving forward. Health workers appear not to have been adequately catered for in terms of logistic supplies to maintain safety protocols, and this contributed to avoidable increased infection among them.

A positive success factor has been weekly joint updates by the Ministry of Information and the Ghana Health Service, complimented by nationwide periodic broadcast by the President. There were occasions when unnecessary arguments occurred about what to report, how, quality, and frequency of COVID-19 reporting, depending on access to what information and the person's political hue. Partisan political colouration should be separated from serious public health issues.

Balancing between socio-economic, political, and technical realities is not always easy and requires level-headedness with ability to navigate through many stakeholders with varying interests. Sensitivity to public concerns in an election year might have distorted some of the national response, and possibly things might have been done differently in a non-election year.

An example is navigating through when to lift the lockdown, open international borders, and the requirement to register and conduct elections by the Electoral Commission amid the pandemic.

The closure of the international boarders was an unpleasant but necessary action by the government as the pandemic was ravaging across Europe, Asia, and United States of America. The socio-economic consequences have not been adequately quantified yet. There was a major discussion and pressure, mostly by political activists, on the government to bring stranded Ghanaians home at a time when the country had not adequately put in place systems to track, test, and manage passengers. There were no technical nor epidemiological reasons to have done so and I side with the government's initial reluctance. This conclusion is buttressed by the fact that, since the International airport at Kotoka was reopened from 1^st^ September 2020 up to 15^th^ December 2020, 94,781 passengers passing through the airport has been tested, with 237 of them testing positive (0.5%).^1^ Though the cost involved was high ($150), the results were available within 30 minutes facilitating quick processing and management of passengers.

There is no magic wand to managing pandemics. Even developed countries in Europe and the USA have reeled and continue to reel under, with devastating consequences. High political will supporting competent technical staff that responds to the needs of people are key ingredients to successful preparedness and response to an epidemic or pandemic. Some of the decisions and social interventions put in place might have been based on political grounds, but who says politics (not partisan politics) is not part of public health!

Moving forward, there is the need to strengthen the entire health system with particular focus on building technical capacities in epidemiology, infection prevention and control, infectious disease management specialists, as well as risk communication experts. There must be investment in building well-equipped health facilities across the country, including establishment of a National Infectious Disease Institute. Investment in ensuring access to safe, cost-effective COVID-19 vaccines is also a high priority for the future.
